# Actinomycosis

**Published:** 1900-10-27

**Authors:** 


### ACTINOMYCOSIS.

At the last meeting of the Clinical Society, Sir Dyce Duckworth related a case of acute actinomycosis. The patient was a youth, aged 19, who had been admitted to St. Bartholomew's Hospital complaining of pain and swelling on the lower part of the left side of the chest. A bulging gradually became apparent, which was more prominent in the left hypochondrium. This bulging was partly thoracic and partly abdominal, and seemed to involve the left lobe of the liver. The tumour, which moved to some extent with the diaphragm, was very tender. Various explorations were undertaken, but without benefit.

At the necropsy a softened patch was found near the cardiac end of the stomach, and a probe readily passed through it into a mass of spongy actinomycotic growth in the left lobe of the liver. The growth had evidently spread indiscriminately, involving the liver, the diaphragm, the left lung, and other parts, and there was a subphrenic abscess of some size, containing pus of a yellowish-green colour with numerous dark green or black granules of ray fungus.

In commenting on this case Mr. Rickman Godlee said he had come across fifteen cases of the disease, and he mentioned one in which the earliest symptoms pointed to pleurisy. This was followed by empyema, from which pus was evacuated. Soon afterwards an abscess appeared in the forearm, and finally another over the front of the sternum. In the latter position a tumour was found after death in which the ray fungus was discovered.

Another case which he mentioned was one of empyema which had resisted treatment and in which subsequently a tumour appeared in the abdomen. This was incised and scraped, and large doses of iodide of potassium were given, and the patient made a good recovery.

Mr. W. G. Spencer also described a case in which recovery had taken place under scraping and the use of large doses of iodide of potassium. The disease started as a swelling in the neck and soon produced a large, brawny induration in that region. The mass was found to protrude into the pharynx. Several sinuses with ragged bluish edges formed in the neck which contained only a small amount of pus, but a large amount of spongy material which gave rise to extreme hæmorrhage. In two months the patient became very much worse. The sinuses were scraped and iodide of potassium was given, but no improvement took place till a month later, when the iodide was given in doses of 40 grains three times a day. Then the constitutional symptoms improved and the sinuses rapidly closed and, in the end, the patient got quite well, though many scars remained in his neck. Mr. Spencer thought that the disease was much more common in cattle than was generally supposed, and that in some cases where it showed signs of its presence the animals were treated with iodide of potassium and then fattened up and sold for meat, and he considered it an important question whether such proceedings should be allowed. In Germany such animals were slaughtered and not allowed to be used for food.

